# Pyrrolizidine alkaloids cause cell cycle and DNA damage repair defects as analyzed by transcriptomics in cytochrome P450 3A4-overexpressing HepG2 clone 9 cells

**DOI:** 10.1007/s10565-021-09599-9

**Published:** 2021-04-21

**Authors:** Sara Abdelfatah, Janine Naß, Caroline Knorz, Sabine M. Klauck, Jan-Heiner Küpper, Thomas Efferth

**Affiliations:** 1 Department of Pharmaceutical Biology, Institute of Pharmaceutical and Biomedical Science, Johannes Gutenberg University, Mainz, Germany; 2 Division of Cancer Genome Research, German Cancer Research Center (DKFZ), German Cancer Consortium (DKTK), National Center for Tumor Diseases (NCT), Heidelberg, Germany; 3 Institute of Biotechnology, Brandenburg University of Technology Cottbus-Senftenberg, Senftenberg, Germany

**Keywords:** Herbal products, Food safety, Pyrrolizidine alkaloids, Systems biology, Transcriptomics, Toxicology

## Abstract

Pyrrolizidine alkaloids (PAs) are a large group of highly toxic chemical compounds, which are found as cross-contaminants in numerous food products (e.g., honey), dietary supplements, herbal teas, and pharmaceutical herbal medicines. PA contaminations are responsible for serious hepatotoxicity and hepatocarcinogenesis. Health authorities have to set legal limit values to guarantee the safe consumption of plant-based nutritional and medical products without harmful health. Toxicological and chemical analytical methods are conventionally applied to determine legally permitted limit values for PAs. In the present investigation, we applied a highly sensitive transcriptomic approach to investigate the effect of low concentrations of five PAs (lasiocarpine, riddelliine, lycopsamine, echimidine, and monocrotaline) on human cytochrome P450 3A4-overexpressing HepG2 clone 9 hepatocytes. The transcriptomic profiling of deregulated gene expression indicated that the PAs disrupted important signaling pathways related to cell cycle regulation and DNA damage repair in the transfected hepatocytes, which may explain the carcinogenic PA effects. As PAs affected the expression of genes that involved in cell cycle regulation, we applied flow cytometric cell cycle analyses to verify the transcriptomic data. Interestingly, PA treatment led to an arrest in the S phase of the cell cycle, and this effect was more pronounced with more toxic PAs (i.e., lasiocarpine and riddelliine) than with the less toxic monocrotaline. Using immunofluorescence, high fractions of cells were detected with chromosome congression defects upon PA treatment, indicating mitotic failure. In conclusion, the tested PAs revealed threshold concentrations, above which crucial signaling pathways were deregulated resulting in cell damage and carcinogenesis. Cell cycle arrest and DNA damage repair point to the mutagenicity of PAs. The disturbance of chromosome congression is a novel mechanism of Pas, which may also contribute to PA-mediated carcinogenesis. Transcriptomic, cell cycle, and immunofluorescence analyses should supplement the standard techniques in toxicology to unravel the biological effects of PA exposure in liver cells as the primary target during metabolization of PAs.

**Graphical abstract:**

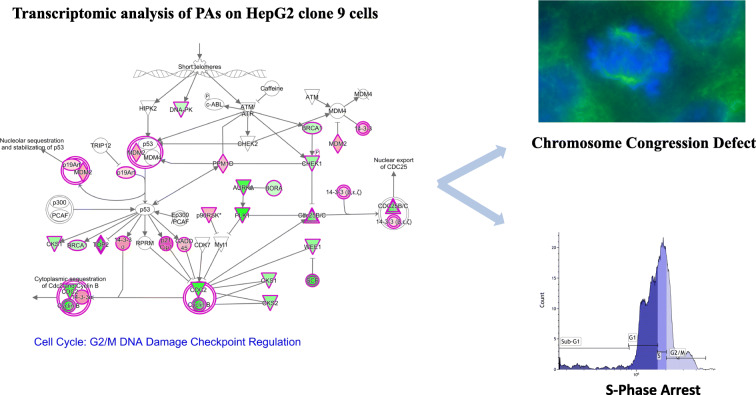

## Introduction

Phytochemicals from vegetable diet and pharmaceutical products are usually considered safe. However, the general assumption about the safety of natural products is a misconception. Nutritional products and herbal medicines can be contaminated with highly toxic compounds, including pyrrolizidine alkaloids (PAs). They are hepatotoxic and carcinogenic even at very low doses, and their intake has to be minimized (Bode and Dong 2015; Schrenk et al. 2020). This is a large class of more than 600 natural compounds, occurring in estimated 6000 plant species, representing 2% of all flowering plants. They can be found in species of various plant families, e.g., Apocyanaceae, Asteraceae (Compositae), Boraginaceae, Fabaceae, Leguminosae, Ranunculaceae, and Scophulariaceae (Tasca et al. 2018). PAs include a wide variety of chemical structures. The basic structure consists of a necine base coupled with one or two necine acids by ester linkages. They are classified based on the necine saturation either as fully saturated or 1,2-unsaturated PAs (Schrenk et al. 2020). PAs play a major role as plant defense mechanisms against fungal or bacterial infections and against attacks by mammalian herbivores and insects (Hartmann, 1994; Hartmann 1999).

Importantly, PAs were detected in several food products (e.g., honey, black tea, green tea, baby teas, and herbal infusions) as well as in medicinal tea products (e.g., peppermint, chamomile, or fennels). Most plant preparations are contaminated with minor amounts of PAs as cross-contamination during harvest (Bodi et al. 2014; Schulz et al. 2015). Considerable health problems arise due to the occurrence of PAs in herbal teas for infants and pregnant or lactating women. In Germany, a recent study showed that 86% of tested herbal tea sampled intended for babies or mothers were contaminated with PAs (Mädge et al. 2015). In principle, PAs can get into meat, milk, or eggs through contaminated feedstuffs (de Nijs et al. 2017; Mulder et al. 2016). There is no doubt that precautions have to guarantee that herbal products usually consumed by babies or lactating mothers do not exhibit harmful toxicities to babies (BfR Bundesinstitut für Risikobewertung 2020)

PAs cause acute liver failure by veno-occlusive disease (VOD) as result of hepatic endothelial damage (Stegelmeier et al. 1999; Chojkier 2003; Rubbia-Brandt 2010). Toxicity related to accidental consumption of 1,2-unsaturated PA-containing food has been documented in several countries. Severe food poisoning is accompanied by hepatic sinusoidal, rapid liver failure, and hemorrhagic necrosis. PAs also exert chronic toxicity due to long-term consumption, and PA accumulation in the body leads to hepatic cirrhosis, pulmonary hypertension, congenital abnormalities, and hepatic cancer (Edgar et al. 2015). PAs activate several signaling pathways that are related to hepatocarcinogenesis (Fu 2017). Complications caused by liver toxicity can be limited by monitoring long-term use of PA-containing products, and eventually immediate discontinuation of their intake (Neuman et al. 2015). Toxicity studies should consider the differences between PAs. Special experimental models using physiologically based kinetic (PBK) modeling approaches were applied to convert the in vitro toxicity results in primary hepatocytes into in vivo dose response curves (Chen et al. 2018).

Bioactivation of PAs by cytochrome P450 monooxygenases (especially the CYP3A4 isoenzyme) into toxic metabolites is responsible for their hepatotoxic effects, and cells lacking CYP activity did not reveal genotoxic PA effects (Ruan et al. 2014; Ebmeyer et al. 2019; Hessel-Pras et al. 2020). In cultured primary human hepatocytes, the activity of CYP enzymes was reduced by up to 90% compared to human liver (Morel et al. 1990; Rodríguez-Antona et al. 2002; Elaut et al. 2006).

Therefore, we used CYP3A4-overexpressing HepG2 clone 9 cells (Herzog et al. 2015) in the present study and exposed them with five PAs (lasiocarpine, riddelliine, lycopsamine, echimidine, and monocrotaline). We aimed to determine at which threshold PAs exert biological activity at the molecular level. For this reason, we applied highly sensitive transcriptomic analyses to identify affected signaling pathways depending on the dose applied. Our analyses add to the chemical-analytical determination of PA concentration in plant-derived products by using transcriptomic analyses to unravel biological mechanisms responsible for the toxicity of PAs.

## Material and methods

### Cell line and culture conditions

CYP3A4-overexpressing HepG2 clone 9 cells are established as previously described (Herzog et al. 2015). Cells were grown in DMEM medium (Life Technologies, Schwerte, Germany) at 37 °C and 5% CO_2_ in a humidified incubator. DMEM media were supplemented with heat-inactivated 10% fetal bovine serum (FBS), 100 U/mL penicillin, and 100 μg/mL streptomycin (Invitrogen, Darmstadt, Germany). Treatment with 3 μg/mL blasticidin was performed to maintain the selection of transfected *CYP3A4*-overexpressing HepG2 clone 9 cells.

### Chemicals

**Fig. 1:**
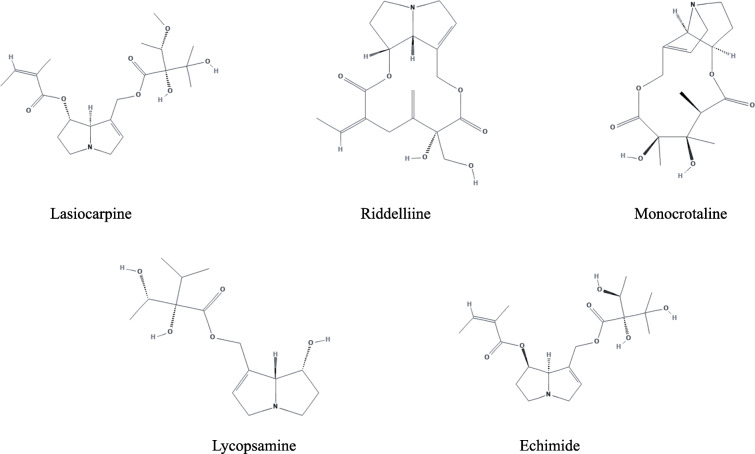
Chemical structures of PAs used in this study: lasiocarpine, riddelliinee, monocrotaline, lycopsamine, and echimidine

Lasiocarpine (purity ≥ 95.0%, HPLC), riddelliine (purity ≥ 90.0%, HPLC), lycopsamine (purity ≥ 95.0%, HPLC), echimide (purity ≥ 90.0%, HPLC), and monocrotaline (purity ≥ 95.0%, HPLC) were purchased from Phytolab (Vestenbergsgreuth, Germany) (Fig. 1).

### Cell treatment with PAs for transcriptomic analyses

Cytochrome P450 3A4-overexpressing HepG2 clone 9 cells were seeded on a culture plate (60 mm) at a density of 700,000 cells/plate. Medium was changed every 24 h. At day 6, medium was removed and cells were trypsinized and washed with PBS. Cells were treated with lasiocarpine, riddelliine, lycopsamine, echimide, or monocrotaline at different concentrations for 24 h. Treatment doses were chosen according to a previous study on the toxicity of these PAs (Gao et al. 2020). DMSO treatment was used as negative control. Duplicates were used for RNA extraction.

### RNA extraction

Total RNA was extracted using InviTrap spin Universal RNA Mini kit (STRATEC Molecular, Berlin, Germany) according to the manufacturer’s instructions. Measurement of RNA concentrations was done using the nanodrop spectrophotometer (Nanodrop Technologies, Thermo Fisher, Dreieich, Germany). The Genomics and Proteomics Core Facility at the German Cancer Research Center (DKFZ, Heidelberg, Germany) performed quality control of RNA, probe labeling, and microarray hybridization of treated and control samples. Each sample was assessed in duplicate. A detailed protocol has been previously described (Zhao et al. 2015).

Briefly, 1 μg total RNA was used for complementary DNA (cDNA) synthesis. Then, MessageAmp™ II aRNA Amplification kit (Ambion, Inc., Austin, TX, USA) was used for amplification/labeling to synthesize biotin-labeled cRNA. Illumina’s recommended sample labeling procedure based on the modified Eberwine protocol was used for preparation of Biotin labeled cRNA samples for hybridization on Illumina Human HT-12 BeadChip arrays.

Then, TotalPrep™ RNA Amplification Kit (Life Technologies, Darmstadt, Germany) was used to purify the cRNA. Hybridization was done following manufacturer instructions. Microarray scanning was performed using Illumina® BeadStation array scanner (Illumina, San Diego, CA, USA). Setting adjusted to a scaling factor of 1 and PMT settings at 430. Data of each sample were extracted, and outliers were removed followed by calculation of mean average signal and standard deviation of each probe. Data were normalized using quantile normalization algorithm.

### Differential gene expression analysis

Chipster software (http://chipster.csc.fi/) was used for differential gene expression analysis between sample and control group. First, data normalization was done using RMA normalization method. Then, genes were filtered on the basis of their standard deviation from the gene mean (percentage to filter out 0.5). All genes with at least one missing value were removed. Differential gene expression analysis between sample and control group was done with two group tests using empirical Bayes *t*-test with a *p*-value threshold 0.05.

### Pathway analysis

Ingenuity Pathway Analysis software (IPA) (http://www.ingenuity.com/) was used for determination of most significantly enriched pathways in each dataset. We also applied an important feature of IPA, the compare analysis which allows analysis from different experimental groups, in order to identify similarities, differences, and trends among the test samples. We compared different concentration effects of each of PAs tested and generated heatmaps of canonical pathways and upstream regulators. We also visualized gene heatmaps for a complete insight into deregulated gene expressions (upregulation or downregulation) in our tested samples. GraphPad Prism software (GraphPad Software, Inc., La Jolla, CA, USA) was used to illustrate gene expression fold changes.

### Cell cycle analysis

HepG2 clone 9 cells were treated with different concentrations of the 5 PAs for 24 h. Then, cells were fixed using 80% cold ethanol and incubated overnight at −20 °C. Cells were then washed twice with PBS and resuspended in 500 μL PBS and stained with propidium iodide (50 μg/mL, Sigma-Aldrich, Taufkirchen, Germany). After 15 min incubation, the measurements were performed using a BD Accuri™ C6 flow cytometer (Becton-Dickinson, Heidelberg, Germany). DMSO-treated cells were used as negative control.

### Immunofluorescence microscopy

HepG2 clone 9 cells were seeded and treated with lasiocarpine (2.5 or 5 μM), riddelliine (15 or 25 μM), monocrotaline (75 or 150 μM), lycopsamine (75 or 150 μM), and echimide (12.5 or 25 μM) for 24 h. DMSO-treated cells were used as negative control. Cells were fixed using 3.7% paraformaldehyde for 30 min at room temperature and washed with PBS. Then, blocking was performed using 5% FBS and 0.3% Triton X-100 in PBS for 1 h. This was followed by staining with primary antibody rabbit α-tubulin antibody (ab52866, Abcam) for 2 h. Cells were washed with PBS, and then a secondary antibody (goat anti-rabbit IgG H&L, Alexa Fluora 488) was added for 1 h. Finally, cells were stained with 2 mg/mL 40,6-diamidino- 2-phenylindole (DAPI) (Sigma-Aldrich). Mounting medium Fluoromount-Gs (SouthernBiotech, Birmingham, AL, USA) was added before microscopy detection. For fluorescent imaging, we used an EVOS SL digital inverted microscope (Life Technologies).

## Results

### Transcriptomic analyses

**Fig. 2:**
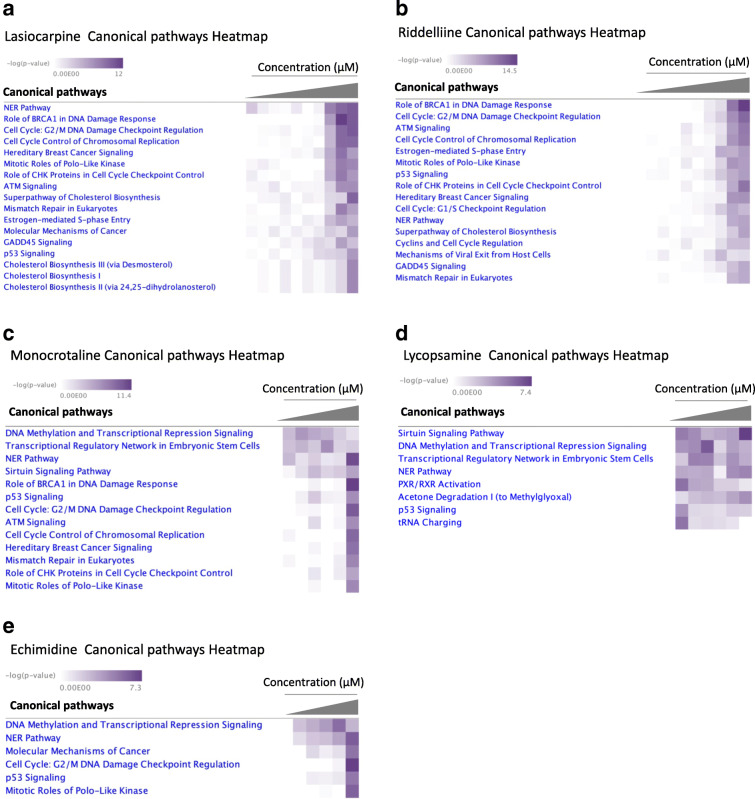
Canonical pathway enrichment analysis. Heatmaps produced by IPA visualize enriched canonical pathways significantly affected in HepG2 clone 9 cells treated with PAs. Compare analysis of different concentrations of: **a** lasiocarpine, 0.01, 0.025, 0.05, 0.1, 0.25, 0.5, 1, 2.5, 5, and 25 μM; **b** riddelliine, 0.25, 0.5, 1, 2.5, 5, 7.5, 15, 25, and 50 μM; **c** monocrotaline, 1, 5, 25, 75, 150, and 300 μM; **d** lycopsamine, 1, 5, 25, 75, 150, and 300 μM; **e** echimidine, 1, 2.5, 5, 12.5, and 37.5 μM. Purple blocks indicate *p*-value scores (Fisher’s exact test) that measure the significance of the pathway’s association with the dataset; white blocks indicate no significant correlation. -Log10 (*p*-value) > 5

We tested five PAs (lasiocarpine, riddelliine, lycopsamine, echimidine, and monocrotaline) on *CYP3A4*-overexpressing HepG2 clone 9 cells and used transcriptomics, in order to detect the pathways that were significantly dysregulated at different concentrations compared to the untreated control. We performed analyses of transcriptomic data using IPA tool for comparing different datasets represented as different concentrations. The canonical pathway analyses showed that the affected pathways significantly varied between different concentrations (Fig. 2). For lasiocarpine, we used the following concentrations: 0.01, 0.025, 0.05, 0.1, 0.25, 0.5, 1, 2.5, 5, and 25 μM. We observed a significant increase in canonical pathways responsible for DNA damage repair and cell cycle regulation at concentrations above 2.5 μM in a dose-dependent manner as represented with dark purple color in Fig. 2a. High doses also significantly altered the expression of the cholesterol biosynthesis signaling pathway as the transcriptomic analysis of the canonical pathways upon riddelliine treatment is shown in Fig. 2b. Signaling pathways of DNA damage repair and cell cycle regulation were also significantly altered by riddelliine treatment, and the effect was significant at the two highest concentrations (25 and 50 μM).

We performed transcriptomics on samples treated with monocrotaline at different concentrations (1, 5, 25, 75, 150, and 300 μM). Two signaling pathways were significantly expressed across all different concentrations (Fig. 2c): (1) DNA methylation and transcriptional repression signaling and (2) transcriptional regulatory network in embryonic stem cells. Signaling pathways responsible for DNA damage repair and cell cycle regulation were only significant at a very high, toxic concentration of 300 μM.

The IPA signaling pathway analysis of lycopsamine-treated cells showed significant effects on the sirtuin signaling pathway, DNA methylation and transcriptional repression signaling, and PXR/RXR activation pathway (Fig. 2d). Significant effects on cell cycle signaling pathways were not found at any of the concentrations used.

Echimidine was tested at concentrations of 1, 2.5, 5, 12.5, and 37.5 μM, respectively. As shown in Fig. 2e, only the highest concentration exhibited significant activation of cell cycle signaling pathways, while similar effects were not seen at lower concentrations. DNA methylation and transcriptional repression signaling was significantly altered across the whole concentration range compared to untreated control.

According to these results, different PAs revealed various effects either depending on their molecule structure itself or the concentration that could exert cytotoxic and carcinogenic effects.

### Expression of DNA damage and cell cycle genes

**Fig. 3:**
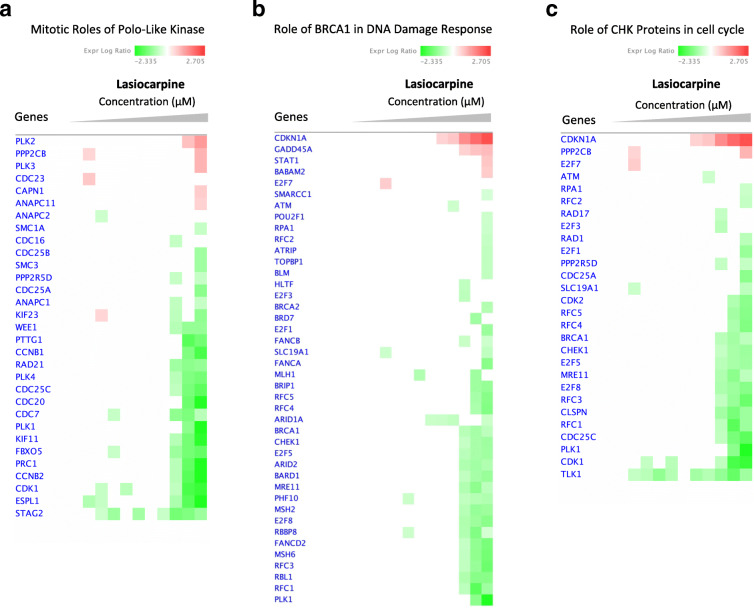
Heatmaps created from compare analysis represent gene expression levels in different pathways of HepG2 clone 9 cells treated with lasiocarpine. **a** Mitotic roles of Polo-like kinase pathway; **b** role of BRCA1 in DNA damage response pathway; **c** role of CHK proteins in cell cycle pathway. Red blocks, upregulated genes; green blocks, downregulated genes; white blocks, not present in the data set

Upon lasiocarpine treatment, changes in gene expression ere mainly observed in signaling pathways related to cell cycle regulation and DNA damage repair as represented by heatmap analyses. The heatmap analyses highlighted overexpressed genes (red-labeled symbols) and downregulated genes (green-labeled symbols), where the intensity of the color represented the degree of significance (Fig. 3). Most genes were significantly altered at high concentrations (2.5, 10, or 25 μM), unlike lower concentrations, where no significant differences in gene expression were observed.

**Fig. 4:**
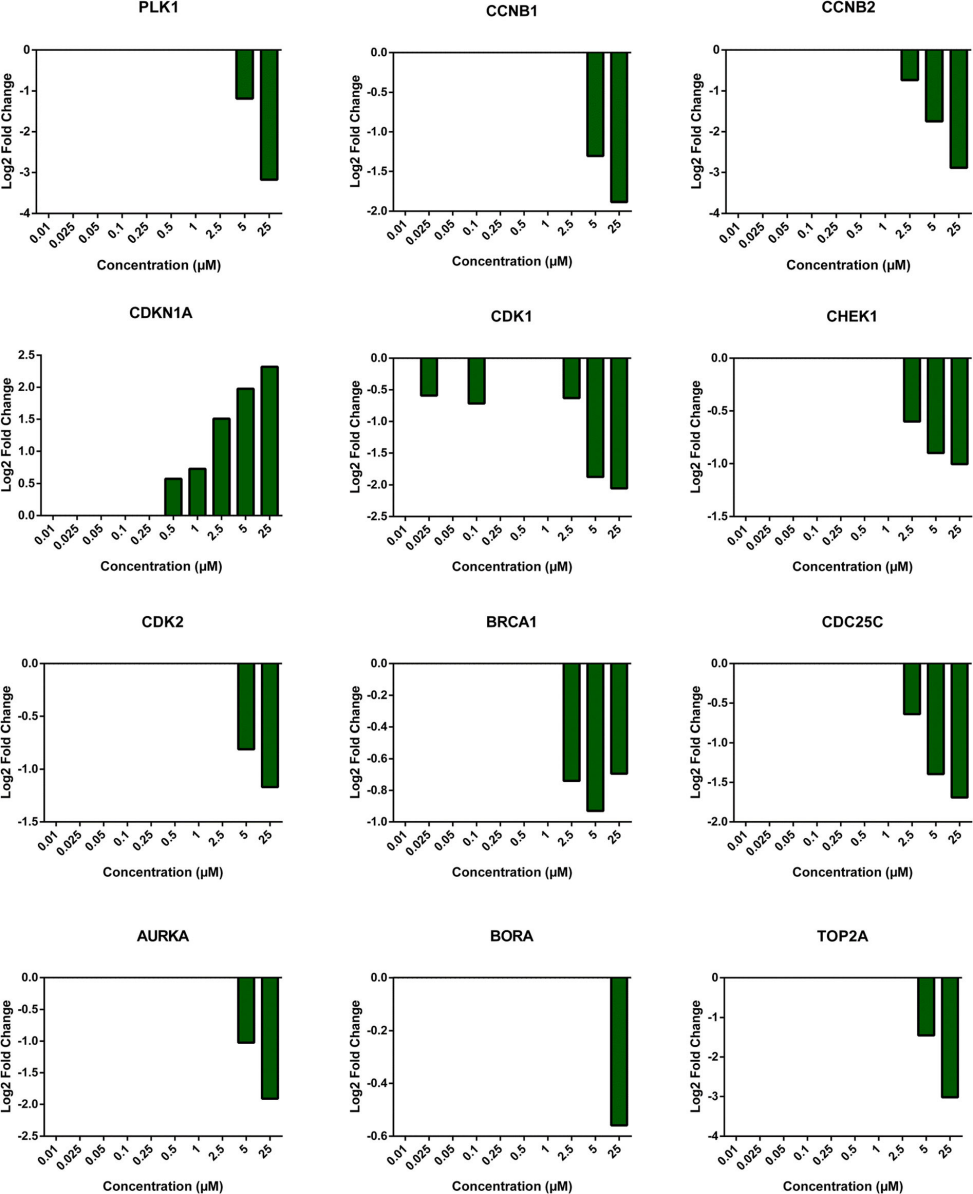
Graphs showing Log2 fold changes of significantly regulated genes of HepG2 clone 9 cells treated with different concentrations of lasiocarpine

For further analysis, we selected 12 genes involved in the regulation of cell cycle and DNA damage repair, i.e., *PLK1*, *CCNB1*, *CCNB2*, *CDKN1A*, *CDK1*, *CHEK1*, *CDK2*, *BRCA1*, *CDC25C*, *AURKA*, *BORA*, and *TOP2A*. The fold-change expression of these genes was compared to untreated control across different lasiocarpine concentrations. As displayed in Fig. 4, the genes were 1-to 3-fold downregulated at 5 and 25 μM, which reflected a role of lasiocarpine in the downregulation of cell cycle signaling.

**Fig. 5:**
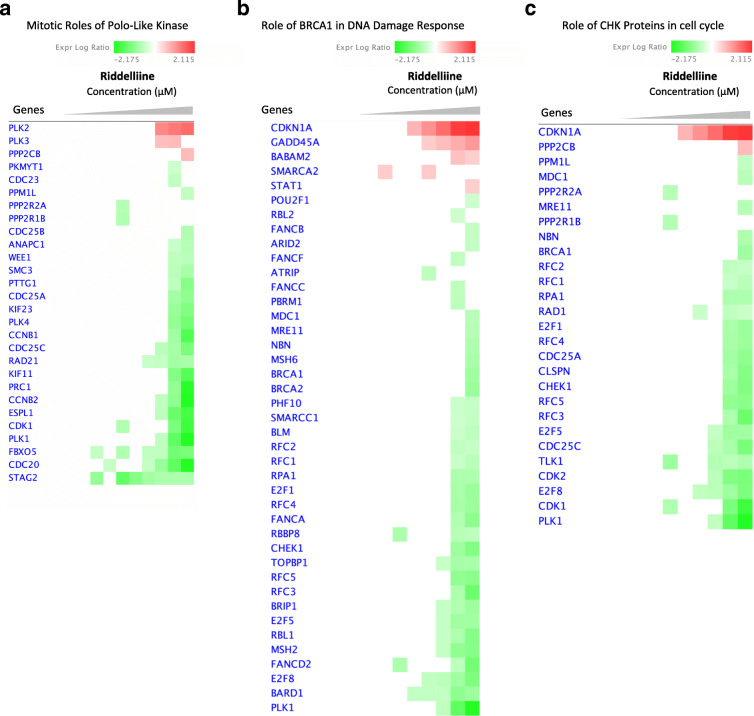
Heatmaps created from compare analysis represent gene expression levels from different pathways of HepG2 clone 9 cells treated with riddelliine. **a** Mitotic roles of Polo-like kinase pathway; **b** role of BRCA1 in DNA damage response pathway; **c** role of CHK proteins in cell cycle pathway. Red blocks, upregulated genes; green blocks, downregulated genes; white blocks, not present in the data set

**Fig. 6:**
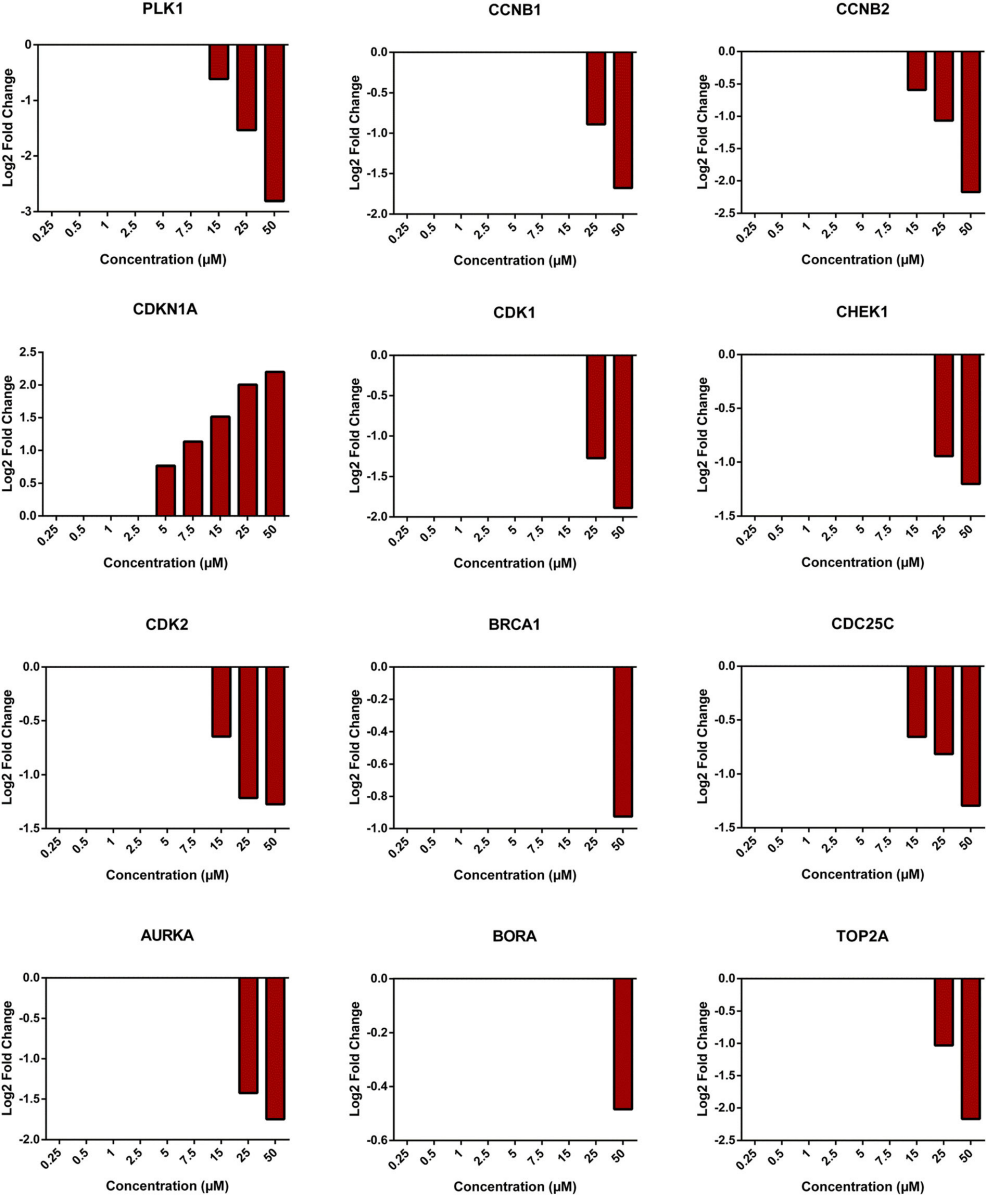
Graphs showing Log2 fold changes of significantly regulated genes of HepG2 clone 9 cells treated with different concentrations of riddelliine

Gene expression analyses were also performed for signal transduction pathways upon treatment with various concentrations of riddelliine. Most genes were only significantly up- or downregulated at the high doses (25 and 50 μM, Fig. 5). The expression ratios of the selected genes showed 1- to 2-fold downregulation at 25 and 50 μM riddelliine (Fig. 6).

**Fig. 7:**
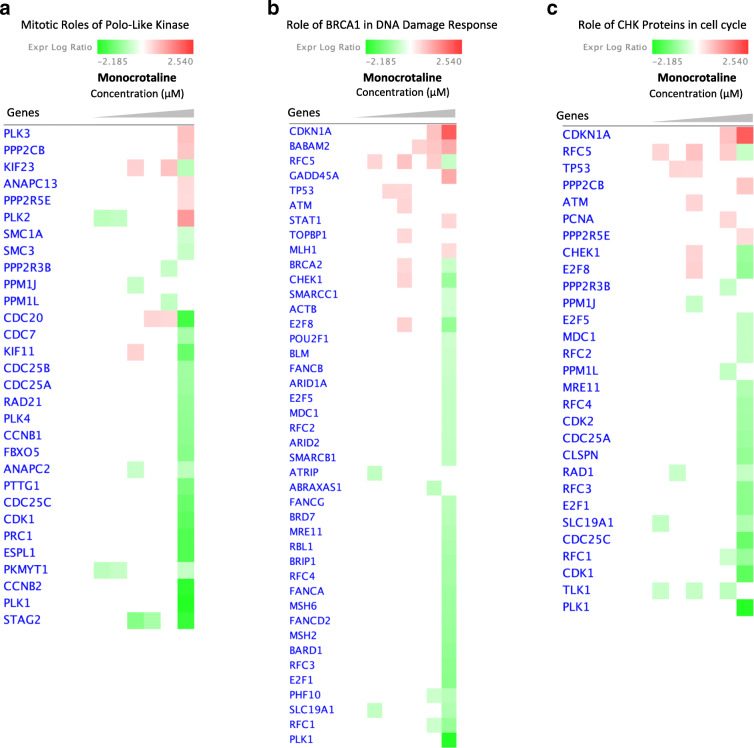
Heatmaps created from compare analysis represent gene expression levels from different pathways of HepG2 clone 9 cells treated with monocrotaline. **a** Mitotic roles of Polo-kike kinase pathway; **b** role of BRCA1 in DNA damage response pathway; **c** role of CHK proteins in cell cycle pathway. Red blocks, upregulated genes; green blocks, downregulated genes; white blocks, not present in the data set

**Fig. 8:**
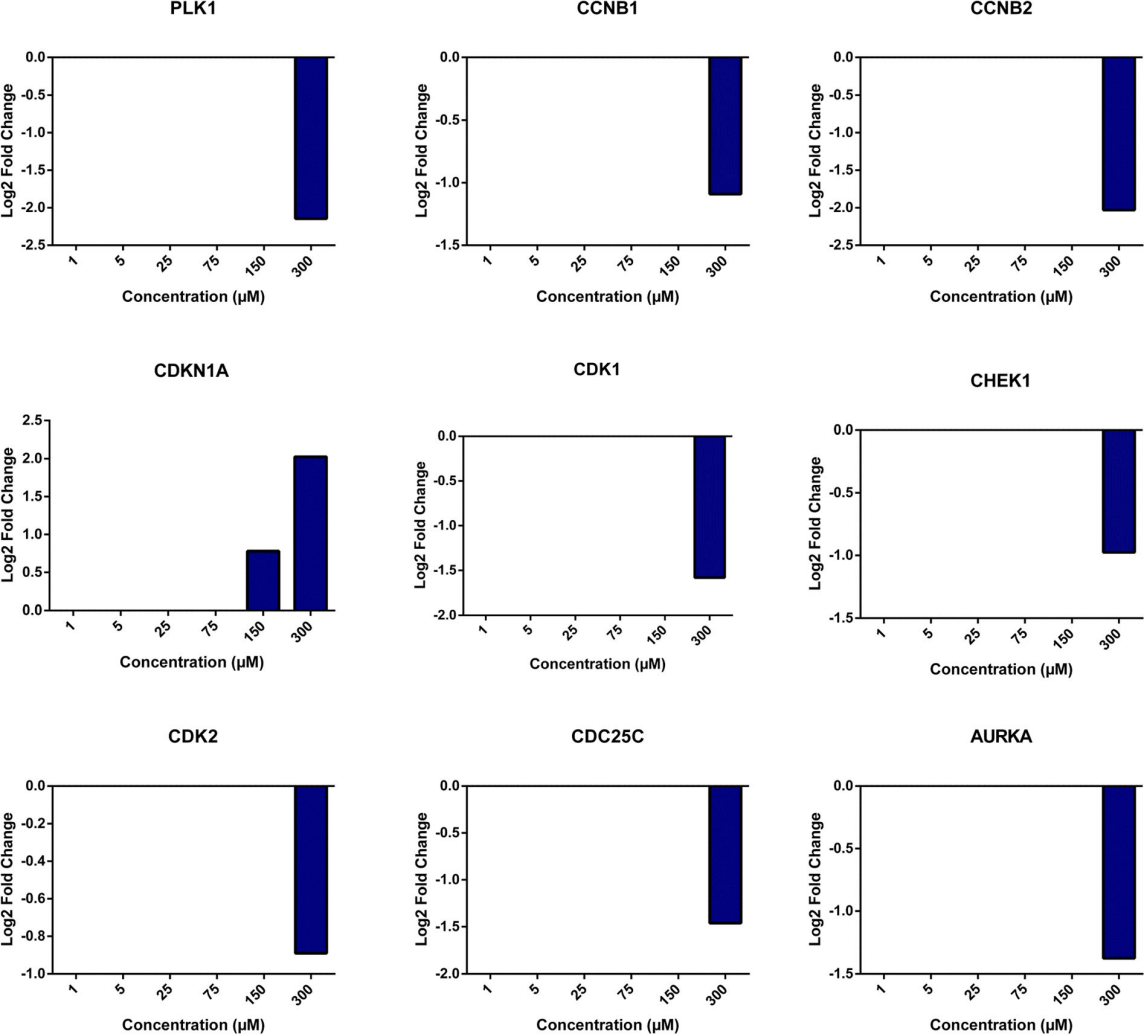
Graphs showing Log2 fold changes of significantly regulated genes of HepG2 clone 9 cells treated with different concentrations of monocrotaline

The altered gene expression in cells treated with monocrotaline is shown in Fig. 7. A significantly changed expression of cell cycle regulating genes was only recorded at the highest concentration tested (300 μM, Fig. 7). The expression ratios of the selected set of genes showed 1.5- to 2-fold downregulation at this concentration (Fig. 8). These results imply fewer toxic effects of monocrotaline compared to lasiocarpine and riddelliine.

**Fig. 9:**
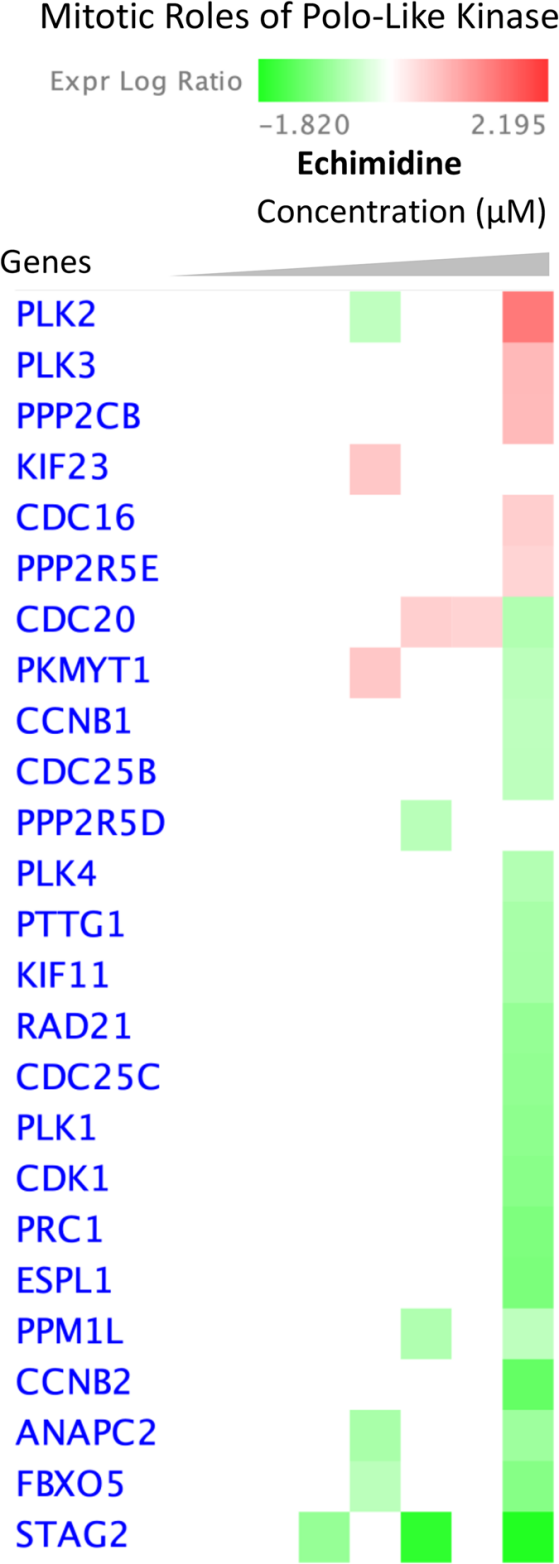
Heatmap represents gene expression of HepG2 clone 9 cells treated with echimidine. Compare analysis showing levels for genes in mitotic roles of Polo-like kinase pathway. Red blocks, upregulated genes; green blocks, downregulated genes; white blocks, not present in the data set

**Fig. 10:**
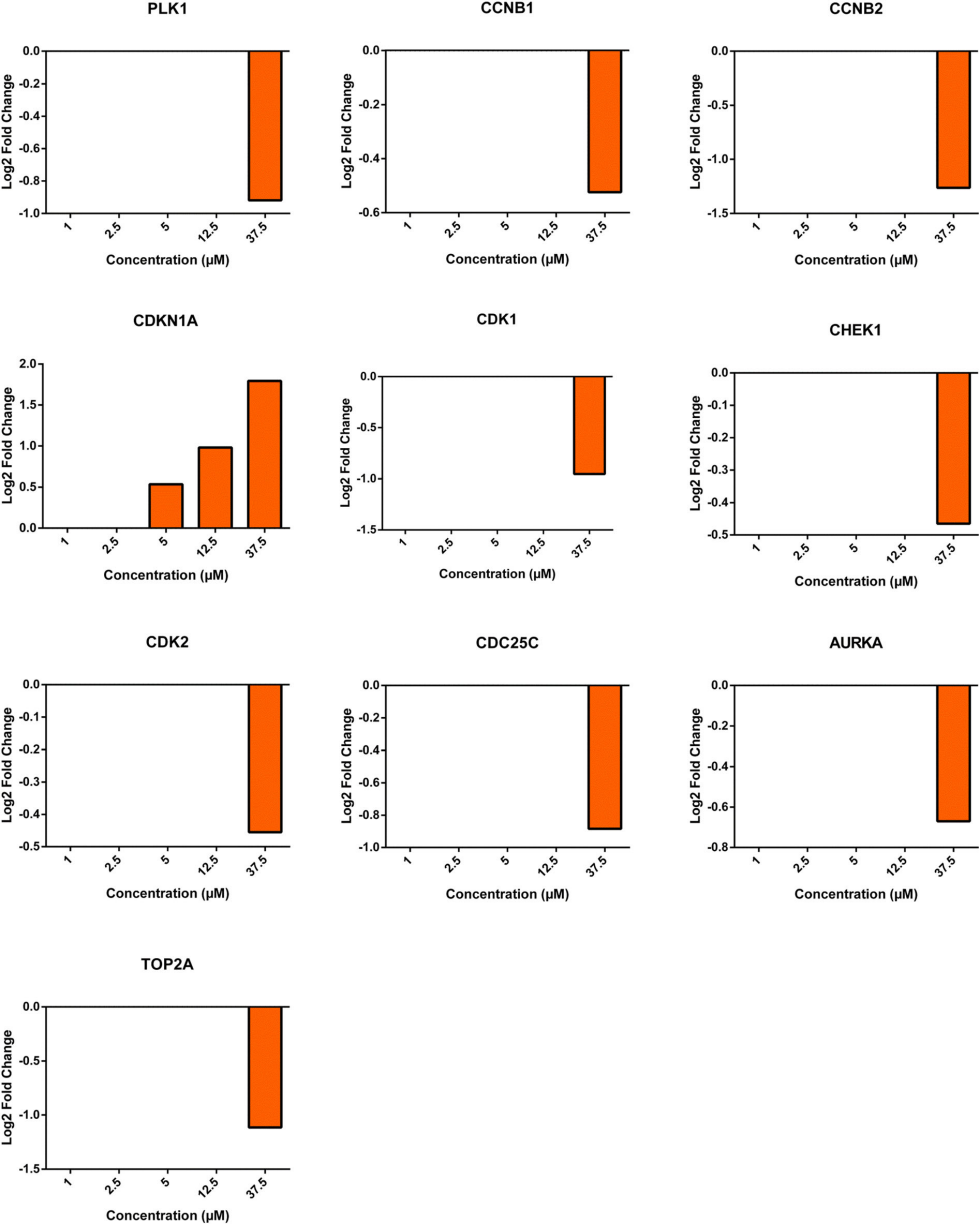
Graphs showing Log2 fold changes of significantly regulated genes of HepG2 clone 9 cells treated with different concentrations of echimidine

Cells treated with echimidine showed significantly changed expression of cell cycle regulating genes at a concentration of 37.5 μM, but no significant effects were noticed at lower concentrations (Fig. 9). The fold-change of expression of these genes was in a range of 1 compared to untreated control (Fig. 10).

We inspected the list of top up- and down-expressed genes shared among high doses of the 5 PAs with a special concern to lasiocarpine and riddelliine. We found an increase in the expression of genes coding for vanin 1 (*VNN1*), matrix metallopeptidase 7 (*MMP7*), cathepsin E (*CTSE*), laminin subunit α3 (*LAMA3*), cytoglobin (*CYGB*), sulfatase 2 (*SULF2*), and keratin 23 (*KRT23*) as well as a downregulation of the gene coding for H2B clustered histone 14 (*HIST1H2BM*). These genes are not involved in the significantly regulated pathways we found (i.e., cell cycle and DNA damage), but their expression pattern indicated that they might be potential targets for the PAs’ activity. Interestingly, PLK1 was shared by samples treated with each of the five PAs as one of the most downregulated genes, indicating that PLK1 may be a driver target for the subsequent disruption of cell cycle and DNA damage repair signaling cascades.

### Cell cycle analysis

**Fig. 11:**
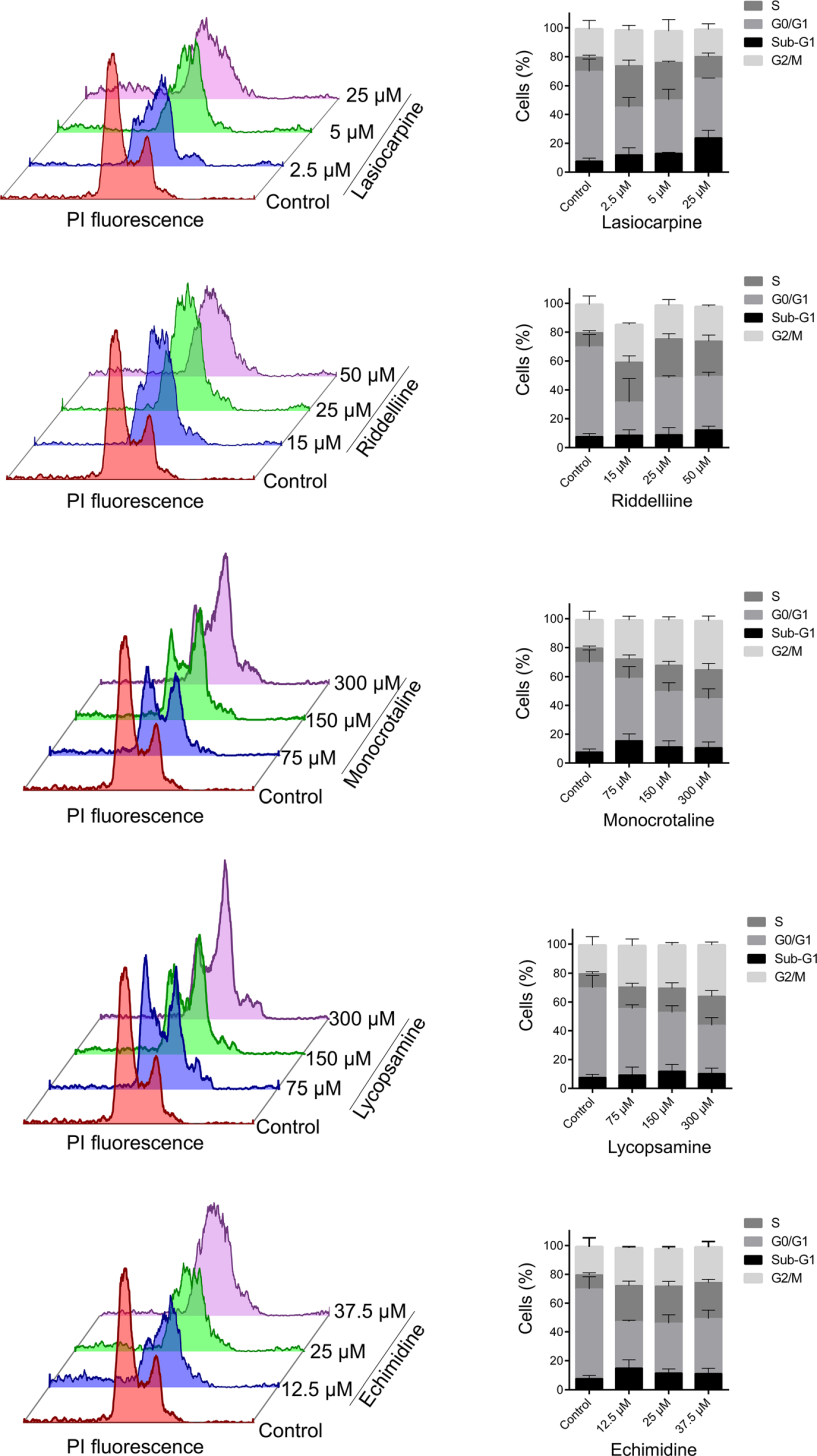
Flow cytometric cell cycle analysis of exponentially growing HepG2 clone9 cells treated with PAs for 24 h with different concentrations. The data are represented as mean and SD of three independent experiments

The results of cell cycle analyses are shown in Fig. 11. Treatment with lasiocarpine revealed a significant increase in the S phase population at 2.5 μM to 28.4 ± 4.1%, at a concentration of 5 μM to 25.6 ±0.9% and with 25 μM to 14.8± 2.5%. The latter decease was explained by an increase of the sub-G1 population (that represent dead cells) to 23.7 ± 5.2%. A similar effect was observed with cells treated with riddelliine. At a concentration of 15 μM, a significant increase of S phase to 27.3 ± 4.5%, and almost similar percentages were noticed at higher concentrations 25 and 50 μM. Treatment with monocrotaline or lycopsamine caused increased S phase fractions for cells treated with 150 or 300 μM and also considerable increases in the G2/M phase. Echimidine also revealed a considerable increase in the S phase population to 25 ± 3% upon treatment with 12.5 μM, and similar effects were found at 25 and 37.5 μM. These results validate the transcriptomic data, which showed changes in the expression of cell cycle regulatory genes.

### Immunofluorescence microscopy

**Fig. 12:**
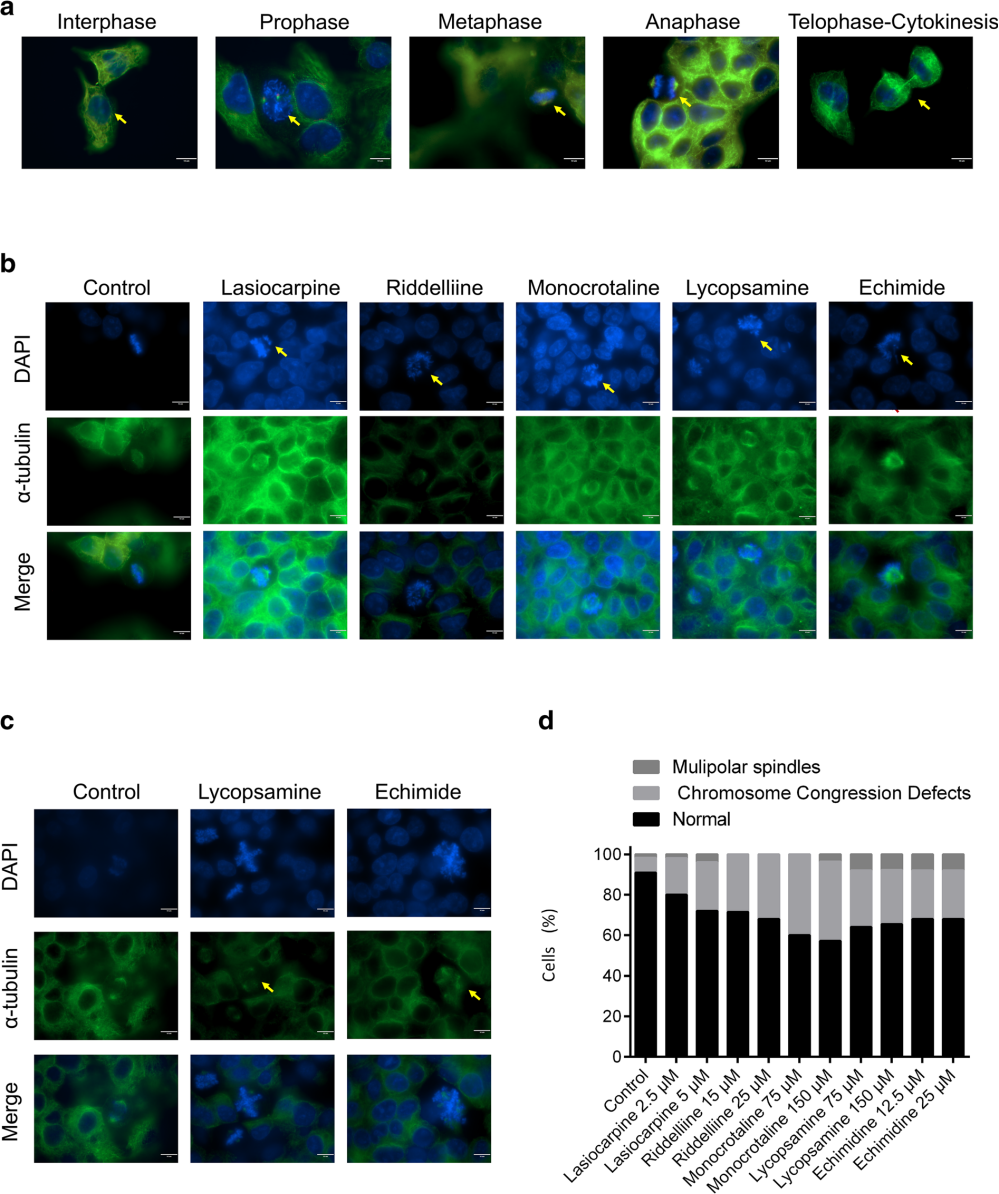
Immunofluorescence analysis of HepG2 clone 9 cells treated with PAs for 24 h. Cells were stained for α-tubulin (green) and DNA (blue). **a** Phases of mitosis for control HepG2 clone 9 cells. **b** Defective chromosome congression after treatment with five PAs. **c** Multipolar spindle formation of HepG2 clone 9 cells treated with lycopsamine and echimide. **d** Percentage of normal mitotic cells and defected cells

Normal HepG2 clone 9 exhibited normal mitotic stages, i.e., interphase, prophase, metaphase, anaphase, telophase, and cytokinesis (Fig. 12a). Based on the immunofluorescence results, treatment with PAs induced chromosome congression defects. As illustrated in Fig. 12b, the five PAs caused a failure of proper alignment of chromosome at the prometaphase and metaphase. Monocrotaline revealed up to 40% chromosome congression defects of mitotic cells at 75 and 150 μM. Treatment with 25 μM riddelliine resulted in 32% defect of chromosome congression.

Another observation on mitotic HepG2 cells clone 9 cells treated with lycopsamine or echimidine was the formation of multipolar spindles (7–8%), where cells failed to form bipolar spindles that mediated proper cell division (Fig. 12c).

## Discussion

The consumption of PA-contaminated foodstuffs and herbal products poses a serious health problem. The main toxic effects are related to liver failure and hepatocarcinogenesis. PAs are mutagenic: they cause mutations in the *TP53* and the oncogene *K-RAS*, and they induce DNA adducts, DNA breaks, and chromosomal damage in vivo and in vitro (Chen et al. 2010).

PA contamination has attracted the attention of regulatory authorities, and legally binding limit values for PAs in different plant-based products were defined to minimize health risks for consumers. Nevertheless, the debate continues both in the scientific community and in the public, whether the limit values for Pas, which have been politically agreed upon and legally fixed by governments, really provide sufficient safety margins in medical terms.

Although it remains difficult to completely remove PAs from food and herbal products, yet their occurrence should be kept to a minimum (BfR 2020). Analytical methods of high sensitivity are required to reliably quantify even small trace amounts of PAs in food products and medicinal herbs (EFSA 2017). As of yet, the gold standard in chemical analytics is high-performance liquid chromatography coupled to mass spectrometry (HPLC-MS), which shows high sensitivity and selectivity (Mulder et al. 2017). However, the disadvantage of this method is that the chemical detection of trace amounts of PAs alone may not sufficiently reflect all relevant biological effects on cells, organs, and whole organisms. Therefore, it remains difficult to draw reliable conclusions about the toxic effects of minimal amounts of toxic substances in contaminated samples. By contrast, the “-omics” technologies allow the identification and quantification of even tiny molecular changes in cells and tissues. This systematic and comprehensive approach allows to also distinguish changes under different conditions, such as disease condition, stress conditions, and seasonal variations. Cell biological changes of even the smallest traces of toxic substances can thus be proven. The “-omics” technologies are therefore basic methods for the new discipline of systemic toxicology (Canzler et al. 2020; Simões et al. 2018).

In this project, we applied transcriptomic analyses for cytochrome P450 3A4-overexpressing HepG2 clone 9 cells treated with five PAs that belong to different PA classes: lasiocarpine, riddelliine, lycopsamine, echimidine, and monocrotaline.

HepG2 clone 9 are stably transfected with *CYP3A4* (Herzog et al. 2015). CYP enzymes (and specifically CYP 3A4) are Phase I detoxification enzymes for many xenobiotic compounds. On the other hand, there are numerous examples, where CYP enzymes (including the isoenzyme 3A4) act not as detoxifiers but as activators; i.e., less toxic compounds get activated by CYP3A4. It is a well-known pharmacological principle that inactive prodrugs get activated to fully active drugs by CYP catalysis (de Montellano 2013). Unfortunately, the same can be true for toxic compounds, where CYP enzymes amplify the toxicity (Rendic and Guengerich 2021). It has been previously published that this is also the case for pyrrolizidine alkaloids. It is widely accepted that CYP enzymes in the liver toxify PAs and that PAs are less harmful without CYP-mediated metabolic activation in the liver (Li et al. 2011; Rutz et al. 2020). This is also true for the CYP3A4 as a major pharmacologically and toxicologically important CYP isoenzyme. It is reported in literature as the main enzyme responsible for metabolization and activation of PAs (Hessel-Pras et al. 2020; Ruan et al. 2014; Ebmeyer et al. 2019). While the human liver expresses CYP enzymes (including CYP3A4) in excessive amounts, primary liver hepatocyte drops down their CYP activity by 90% after 24 h *culturing* (Elaut et al. 2006; Morel et al. 1990; Rodríguez-Antona et al. 2002). Also, many hepatocyte lines (including HepG2) largely lost the expression of CYP enzymes during the immortalization process. It has been documented that HepG2 cells contain only little CYP activity (Ogino et al. 2002; Wilkening et al. 2003). Therefore wild-type HepG2 cells are not suitable as cell models to investigate the harmful effects of PAs. This disadvantage has been overcome by HepG2 cell lines transfected with CYP genes that have been generated and are widely used to study the effects of metabolic activation of xenobiotic compounds. For this reason, we used CYP 3A4-transfected HepG2 cells instead of wild-type HepG2 cells in the present investigation.

Advanced molecular techniques such as transcriptomics provide high sensitivity and hold great promise for the determination of concentration limits of PAs in food products, dietary supplements, and pharmaceutical herbal products. The perturbation of signaling pathways due to deregulated gene expression as consequence of PA exposure adds a new and important dimension in toxicology beyond the conventional chemical detection technology for poison monitoring.

Signaling pathway analyses showed an unambiguous dysregulation of circuits related to DNA damage repair and cell cycle regulation: this included a role of BRCA1 in DNA damage response, G2/M DNA damage checkpoint regulation, cell cycle checkpoint control, a role of Polo-like kinase in mitosis, and a role of CHK proteins in cell cycle and ATM signaling. There is a direct interplay between DNA damage responses and cell cycle regulation mechanisms. Both pathways share common upstream regulators such as p53 and ATM signaling. This link is important to maintain genome stability and to allow cells to undergo prolonged mitotic arrest and thereby to initiate DNA repair upon DNA damage (Shaltiel et al. 2015).

PA treatment significantly inhibited Polo-like kinase 1 (*PLK1*) expression. PLK1 is an important regulator of the M phase of the cell cycle. It activates the mitotic entry through activation of the cyclin-dependent kinase 1 (CDK1)–cyclin B complex and mediates spindle and centromere formation. PLK1 also stabilizes mitotic division and mitotic exit (Jackman et al. 2003; Sanhaji et al. 2014). Transcriptomic analyses also showed a downregulation of *BORA* and *AUKRA*. The proteins encoded by both genes function as complex that regulate PLK1 activation and mitotic entry (Seki et al. 2008).

Another major player in response to DNA damage and regulation of cell cycle progression is checkpoint kinase 1 (*CHEK1*). CHEK1 is activated by phosphorylation and inhibits the activity of cyclin-dependent kinase (CDK). It stabilizes stalled replication forks and suppresses replication origin firing. The function of CHEK1 is well known in cancer cells with defective DNA synthesis machinery (Smits and Gillespie 2015). In our investigation, CHEK1 signaling was significantly inhibited upon treatment with high doses of lasiocarpine, riddelliine, and monocrotaline.

Moreover, the BRCA1 signaling pathway showed a significant response to PA treatment. *BRCA1* is a well-known tumor suppressor gene, which is frequently mutated in breast and ovarian cancers. BRCA1 contributes to genomic stability and mediates responses to DNA damage. It localizes to the DNA breakage site and interacts with chromatin remodeling proteins, which modulate the mending of breakage sites. It is also required for the activity of the cell cycle checkpoint in S and G2/M phases. The function of BRCA1 is regulated by upstream effectors such as ATM, ATR, and Chk2 (Wu et al. 2010).

The dysregulation of DNA damage and cell cycle signaling pathways explains the carcinogenic effects of PAs. This is in accord with the fact that the effects on gene expression were more pronounced upon treatment with the more toxic PAs lasiocarpine and riddelliine compared to the less toxic monocrotaline. On the other hand, lycopsamine did not significantly affect cell cycle or DNA damage regulation circuits. DNA damage and genotoxicity by lasiocarpine and riddelliine have been previously investigated in primary rat hepatocytes and human HepaRG cells as detected by phosphorylation of histone protein H2AX (Chen et al. 2019).

In our investigation, the expression profiles of genes involved in cell cycle and DNA damage repair showed that the carcinogenic effects of PAs were dependent on the concentration. This implicates a toxicity limit of 1 μM for lasiocarpine and 15 μM for riddelliine. The limit value for monocrotaline was much higher (above 150 μM). Our results are corroborated by a previous study on toxicity levels of PAs, where lasiocarpine was the most potent cytotoxic PA among 14 PAs tested in rat hepatocytes (Merz and Schrenk 2016). Cytotoxicity assays reported higher toxicity levels of lasiocarpine and dicyclic esters (except monocrotaline) compared to monoesters on HepG2 clone 9 cells and rat hepatocytes. The latter were more sensitive because of variations in CYP expression (Rutz et al. 2020).

We confirmed the results of transcriptomic analyses by applying cell cycle analyses. Here, the PAs exerted S phase arrest in HepG2 clone 9 cells, which was in line with the results obtained by our transcriptomic experiments. We also investigated the behavior of cells during mitosis by immunofluorescence staining. PA treatment resulted in the abnormal alignment of chromosomes during metaphase. This phenomenon is known as a defect of chromosome congression (Maiato et al. 2017). Besides, multipolar spindle formation was reported upon treatment with lycopsamine (retronecine-type monoester pyrrolizidine alkaloid) and echimidine (retronecine-type open diester PA). Multipolar spindles are responsible for mitosis arrest and failure of cell cycle progression (Bhakta-Guha et al. 2015; Sertel et al. 2011). This reflects that different chemical classes may have different carcinogenic mechanisms.

Gene expression profiling gave a clear input of mechanisms of PAs’ toxicity. Cell cycle distribution and chromosomal alignment defect occurred as result of PA treatment, which further validated the transcriptomic results.

DNA lesions lead to cell cycle arrest, which provide damaged cells sufficient time to activate the DNA repair machinery or to induce apoptotic cell death. The interplay between cyclins, cyclin-dependent kinases (CDKs), and CDK inhibitors enables the cell not only cell cycle arrest but also the continuation of cell cycle progression after DNA damage has been repaired. Our analyses show that chromosomal alignment defects also occurred upon PA exposure. Correct chromosome congression is necessary for proper chromosome segregation during mitosis. PA-induced defects in chromosome congression may imply that the completion of the mitotic process and the continuation of cell cycle progression is hindered. As molecular regulators of chromosome congression (e.g., Kif18A) are involved in carcinogenesis (Zhang et al. 2010), it is well possible that disturbances of chromosomal congression contribute to the carcinogenic effects of PAs in addition to the mutagenicity of PAs in DNA. To the best of our knowledge, PA-induced defects in chromosome congression are described here for the first time.

Our investigation contributes to the ongoing risk assessment discussion, whether or not the current limit values should be decreased, since contamination of herbal medicines and food products with PAs continues to be a serious issue regarding safety of these products. Hundreds of PAs have been detected in foods (NTP 2003; EFSA 2017). The European Food Safety Authority (EFSA) Panel on Contaminants in the Food Chain (EFSA CONTAM Panel) proposed 17 PAs to be monitored in food products together with the recommendation to further increase PA testing (EFSA 2017). The European Union generally recommends that the exposure to genotoxic and carcinogenic substances should be as low as reasonably achievable (ALARA principle). The German Federal Institute for Risk Assessment (BfR) assessed the toxicity and health risk of PAs in food products and concluded that doses of daily intake of 1,2-unsaturated PAs has to be kept as low as possible (BfR 2020). Health authorities are concerned to define legally binding limit values for daily PA intake. According to the German drug law-based monographs (AMG, §5, 7), the daily exposure of 1,2 unsaturated Pas must not exceed 100 μg/person for external and 1 μg/person for internal application for maximal 6 weeks/year. In 2016, the Public Statement of the Committee on Herbal Medicinal Products (HMPC) has recommended a threshold of 1.0 μg/day of PAs as transitional measure for a period of 3 years, after which the threshold should be set to 0.35 μg/day, a level that was originally addressed by the European Food Safety Authority (EFSA). These recommendations have been approved by the German Medicines Agency (BfArM) and report to improve the usual Good Agricultural and Collection Practices (GACP). In 2019, the transitional period for products with levels up to 1.0 μg PAs/day was prolonged for another 2 years (EFSA 2017; HMPC 2016; HMPC 2019). The International Agency for Research on Cancer (IARC) of the World Health Organization (WHO) also classified various PAs as “possibly carcinogenic to humans” (IARC 2002).

The PA contents in plant-based food have been considerably decreased during the past years, which decreased the total PA exposure and thereby health risk of consumers. The PA exposure via herbal medicines, spices, and dietary supplements is less well documented yet.

We think application of transcriptomic analyses in the field of toxicology provides enormous possibilities. The majority of toxic xenobiotics cause mutations or alteration in gene expression levels. Using standard methods, it remains difficult to get a comprehensive insight into the mechanistic processes of toxicity and the affected genes. The power of transcriptomics led to a rapid distribution in many different areas of life science and biomedicine. Transcriptomics is also a core technology, which constituted to the field of toxicogenomics. This new discipline enables to investigate the molecular mechanisms of toxic compounds in its entirety (Waring and Halbert 2002; Waters et al. 2003; de Longueville et al. 2004). It has also been discussed to use toxicogenomic tools for risk assessment in regulatory affairs (Pennie et al. 2004; Chan and Theilade 2005). In herbal medicine, transcriptomics is widely used for the detection of pharmacological mechanisms as well as of safety issues related to the intake of medicinal and nutritional herb preparations. Herbs and herbal preparations consist of complex mixtures of compounds which are frequently difficult to dissect in their biological activity in the human body. Transcriptomics provide a practical approach to generate hypothesis on the multiple modes of action and also to pinpoint the activities of toxic compounds and to elucidate mechanisms leading to the toxicity of herbal products (Tong et al. 2003; Guo et al. 2010; Thompson 2010). There are also some examples where toxicogenomics has been applied in the context of pyrrolizidine alkaloids. A carcinogenesis-related gene expression profile was detected upon treatment with a comfrey extract (*Symphytum officinale* L.) (Guo et al. 2007). Comparable results have been obtained in experiments with isolated pyrrolizidine alkaloids. Genes were differentially regulated in riddelliine-treated rats, which have been assigned by Ingenuity pathway analysis to generally tumor-related mechanisms such as cell death, cellular movement, cell-to-cell signaling and cellular growth and proliferation (Guo et al. 2007; Mei et al. 2007). Treatment of primary human hepatocytes with echimidine, heliotrine, senecione, or senkirkine led to a set of commonly deregulated genes involved in cell cycle regulation, cell death, and cancer development as well as the activation of transcription factors, e.g., TP53, MYC, NF-kB, and NUPR1 (Luckert et al. 2015). Although the results are partly heterogeneous, these studies are in accordance with our investigation that cancer-related mechanisms are activated by pyrrolizidine.

As yet, chemical analytical methods routinely used to monitor PA contaminations (multiple reaction monitoring transition (MRM) technique, LC-MS/MS). Therefore, we highly propose to implement transcriptomic analyses for the analysis of herbal products, baby teas, and dietary supplements. They represent a convenient and reliable technology to directly determine the biologically relevant PA concentrations in suitable experimental models. Therefore, the safety of plant-based products should not only be governed by chemical-analytical methods, but also by sensitive biological techniques such as transcriptomics to monitor the biological hazards of PAs on liver cells.

## Data Availability

The authors declare that the data supporting the findings of this study are available within the paper. All other data are available from the corresponding author upon reasonable request.

## Abbreviations

PAs: pyrrolizidine alkaloids
EFSA: The European Food Safety Authority
BfR: The German Federal Institute for Risk Assessment
IPA: Ingenuity Pathway Analysis software
*PLK1*: Polo-like kinase 1
*CCNB1*: Cyclin B1
*CCNB2*: Cyclin B2
*CDKN1A*: Cyclin dependent kinase Inhibitor 1A
*CDK1*: Cyclin dependent kinase 1
*CHEK1*: Checkpoint kinase 1
*CDK2*: Cyclin Dependent Kinase 2
*BRCA1*: Breast cancer type 1 susceptibility protein
*CDC25C*: Cell division cycle 25C
*AURKA*: Aurora kinase A
*BORA*: BORA Aurora kinase A activator
*TOP2A*: DNA topoisomerase II α
HMPC: The Public Statement of the committee on Herbal Medicinal Products
BfArM: German Medicines Agency
GACP: Good Agricultural and Collection Practices
VOD: veno-occlusive disease
